# Targeted Phytohormone Profiling Identifies Potential Regulators of Spikelet Sterility in Rice under Combined Drought and Heat Stress

**DOI:** 10.3390/ijms222111690

**Published:** 2021-10-28

**Authors:** Maria Vera Jesus Da Costa, Venkategowda Ramegowda, Sheshshayee Sreeman, Karaba N. Nataraja

**Affiliations:** Department of Crop Physiology, University of Agricultural Sciences, GKVK, Bangalore 560065, Karnataka, India; veradacosta5@gmail.com (M.V.J.D.C.); msshesh1@uasbangalore.edu.in (S.S.); nataraja_karaba@yahoo.com (K.N.N.)

**Keywords:** rice, combined drought and heat stress, phytohormones, flag leaf, spikelets

## Abstract

Rice cultivated under rainfed or semi-irrigated ecosystems is frequently exposed to a combination of drought and heat stress. As a sensitive crop at the reproductive stage, exposure to combined drought and heat stress will have a deleterious effect on yield. In this study, two rice cultivars with contrasting spikelet sterility, AVT2-5315 (low sterility) and AC35027 (high sterility), under combined stress were selected for physiological characterization and phytohormonal profiling at anthesis. Under combined stress, both cultivars did not differ in the physiological parameters such as relative water content, photosynthetic rate, light-adapted chlorophyll fluorescence and biomass, suggesting a similar source activity under stress. However, AVT2-5315 showed better yield due to better pollen and spikelet fertility than AC35027, suggesting its intrinsic tolerance ability under combined stress. Targeted profiling of 15 phytohormones from drought, heat and combined stress-treated flag leaf and spikelet tissues using LC–MS/MS showed increased accumulation of auxins (indole 3-acetic acid and indole 3-butyric acid) in flag leaves and jasmonic acid in spikelets of AVT2-5315, while there was increased accumulation of ethylene in flag leaves and methyl-jasmonate in spikelets of AC35027. Increased accumulation of these hormones correlated with key biosynthetic pathway genes. In the flag leaves, increased accumulation of auxins was correlated with increased transcript levels of *YUCCA-like gene 1* (*OsYUCCA1*) and *fish bone* (*OsFIB*), in AVT2-5315 under combined stress. In AC35027, increased ethylene content was correlated with expression of *1-aminocyclopropane-1-carboxylate synthase 1* (*OsASC1*) and *aminocyclopropane-1-carboxylic acid oxidase 2* (*OsACO2*). Similarly, in spikelets, increased accumulation of jasmonic acid in AVT2-5315 was correlated with expression of *allene oxide cyclase* (*OsAOC*) and *12-oxophytodienoic acid reductase 1* (*OsOPR1*). The mechanism of regulating spikelet sterility by these hormones needs further investigation towards improving rice tolerance to combined stress.

## 1. Introduction

Plants under field conditions are routinely exposed to several unpredictable environmental conditions, leading to reduced growth and yield. Currently, drought and heat stress assume a greater significance due to changing climate and population dynamics. According to the intergovernmental panel on climate change (www.ipcc.ch/2014),the global surface temperature and the amount and pattern of rainfall are likely to become more erratic, subjecting crops to a greater range of environmental stresses. It is predicted that by 2025, nearly 15–20 million hectares of rice-growing areas will suffer from water scarcity [1]. Besides this, average temperatures are expected to rise by 2–3 °C over the next 30–50 years [2]. With these inevitable consequences, it is critical to understand the holistic responses of plants not only under individual drought and heat stresses, but also under combined drought and heat stress towards the development of climate-ready crops.

Rice is the most widely consumed crop worldwide, especially in Asia. Being a high water-demanding crop, it is extremely sensitive to drought and heat stresses, especially at the reproductive stage. Individual drought and heat stresses during anthesis and pollen germination have been shown to drastically reduce yield compared to other growth stages [3]. Heat stress during flowering can cause abnormal anther dehiscence, resulting in low pollen count, asynchronous pollination, reduction in pollen tube growth, a shorter flowering and grain filling period, reduced grain weight, and increased chalkiness [4]. Drought stress during flowering can inhibit panicle exertion from the flag leaf sheath, leading to severe sterility in rice. In addition, under drought, reduced transpiration due to partial or complete closure of stomata leads to a drop in the turgor pressure of reproductive organs such as anthers and stigmas, affecting pollination and fertility. Although rice can maintain normal growth at temperatures ranging from 27 to 32 °C without significant reductions in grain yield, temperatures above 32 °C negatively affect all stages of rice plant growth and development, especially when combined with drought stress [5,6,7,8]. So far, most studies have concentrated on understanding plant responses to individual drought and heat stresses, but under field conditions drought and heat stress frequently co-occur. Recent studies have shown that the physiological, metabolic and molecular responses of plants exposed to combined drought and heat stress are unique from the individual stresses [9,10]. Combined drought and heat stress during flowering has been shown to affect anther dehiscence, pollen number and germination, and spikelet fertility [11]. High air temperatures with severe drought results in high transpiration from spikelets and loss of moisture, needed for pollen swelling—leading to reduced anther dehiscence and subsequent grain set and affecting overall yield [8]. Crop yield is also largely limited by net carbon gain, as photosynthetic carbon metabolism and mitochondrial respiration are extremely sensitive to these stresses, thereby limiting biomass accumulation compared to individual stresses [12,13]. Additionally, a significant reduction in grain yield, seed-setting rate, and harvest index have been reported in rice cultivars under combined stress [7].

Plants have developed several intricate mechanisms to tolerate stress, which include the accumulation of several metabolites, proteins, and phytohormones. Among these, phytohormones are the key regulators of plant growth and development not only under stress, but also under normal conditions [14,15,16]. These phytohormones include auxin (IAA), cytokinin (CK), abscisic acid (ABA), ethylene (ET), gibberellins (GAs), salicylic acid (SA), brassinosteroids (BRs), jasmonates (JAs), and strigolactone (SL). The interplay between these phytohormones in regulating several morpho-physiological, biochemical and metabolic processes not only under stress but also under normal growing conditions is well established [17,18]. The role of these phytohormones in regulating plant responses to individual drought and heat stress has been studied decently in rice [15,19,20,21]. For example, increased ABA resulted in pollen abortion upon drought stress [22]. Increased methyl-jasmonate (Me-JA) levels in rice panicles significantly affected spikelet numbers [23]. Similarly, IAA, GAs, and zeatin+zeatin riboside (Z+ZR) present in young rice panicles increased grain yield [19]. GA has been shown to regulate sugar content in stamens maintaining rice male fertility [22]. On the other hand, under individual heat stress, IAA and GA1 are known to regulate the developmental process of rice reproductive organs during panicle initiation and flowering [15]. CK and ABA have been shown to regulate floret differentiation and the number of rice spikelets [19]. Additionally, IAA, GA, ABA and, CK have been shown to mediate grain weight [15]. Recent findings suggest that BRs in rice panicles promote spikelet development under drought [24] and heat stress [25]. The role of phytohormones under drought and heat has been reviewed extensively [15,19,20,26,27,28,29]. It is evident from individual drought and heat stress studies that phytohormones are critical in regulating both vegetative as well as reproductive growth in rice. So far, there is no study on phytohormones in rice exposed to a combination of drought and heat stress. An understanding of phytohormonal homeostasis and signaling under combined drought and heat stress at the reproductive stage is essential for improving plant performance under the changing climate. In this study, the physiological responses and phytohormone profiles of two rice cultivars differing in spikelet sterility were compared to identify potential phytohormone markers under combined stress.

## 2. Results

### 2.1. Combined Stress Severely Affects the Physiology of Plants in Both the Cultivars

**Figure 1:**
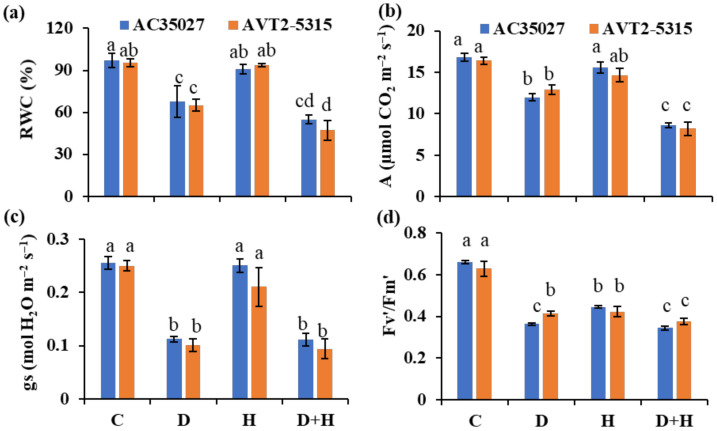
Physiological responses of contrasting rice cultivars under stress. Relative water content (**a**); photosynthetic rate (**b**); stomatal conductance (**c**) and light–adapted maximum quantum efficiency of PSII (**d**) of rice plants treated with control (C), drought (D), heat (H) and combined stress (D+H) at anthesis. Data are mean values ± SE (n = 5). Means with the same letter do not differ significantly at *p* ≤ 0.05.

Two rice cultivars, AC35027 (high sterility) and AVT2-5315 (low sterility), previously identified for their differential stress responses, were exposed to individual drought, heat as well as combined stress during flowering. The weekly maximum temperature ranged from 28–35 °C during the entire growth period (Figure S1a) while during anthesis, the daily maximum temperature ranged from 33–36 °C (Figure S1b). Additionally, the average relative humidity ranged from 33–55% during the entire growth period (Figure S1a), while during anthesis it was 32–39% (Figure S1b). The relative water content (RWC) measured on the third day of stress from the second fully expanded leaf was 70%, 93% and 56% in AC35027 and 68%, 98% and 49% in AVT2-5315 under drought, heat and combined stress, respectively (Figure 1a). In both the cultivars, control and heat-stressed plants showed a similar RWC. Measurement of photosynthetic rate (A) showed a severe reduction by 49–50% in both the cultivars under combined stress (Figure 1b). Under drought, a decrease of 21–29% was observed (Figure 1b). The reduction in stomatal conductance (gs) under combined stress was comparable with drought stress (Figure 1c). Both ‘A’ and ‘gs’ under heat stress were comparable with the control condition. The light-adapted chlorophyll fluorescence (Fv’/Fm’) was similarly affected by all stresses (Figure 1d). Among the cultivars, AVT2-5315 showed a 55% reduction, while AC35027 showed a 66% reduction under drought stress compared to control. Under heat stress, increased transpiration was observed compared to other treatments, indicating transpirational cooling (data not shown). The intrinsic water use efficiency (A/gs) was greater under drought stress, followed by combined stress (Figure S2a). There was no significant difference between heat stress and control plants. Additionally, the effective quantum yield of PSII (ΦPSII) was severely affected under combined stress compared to individual stresses in both the cultivars (Figure S2b). Besides, combined stress resulted in a greater decrease in photochemical quenching (qP) and an increase in non-photochemical quenching (qN; Figure S2c,d). The highest qN was observed in heat-treated plants compared to drought and combined stress as well as the control condition. Together, the results show that combined stress severely affects plant physiological processes, and between drought and heat stress, drought seems to be the dominant stress in the combination.

### 2.2. Intrinsic Tolerance Was the Potential Cause of Better Fertility and Yield in AVT2-5315 under Combined Stress

**Figure 2:**
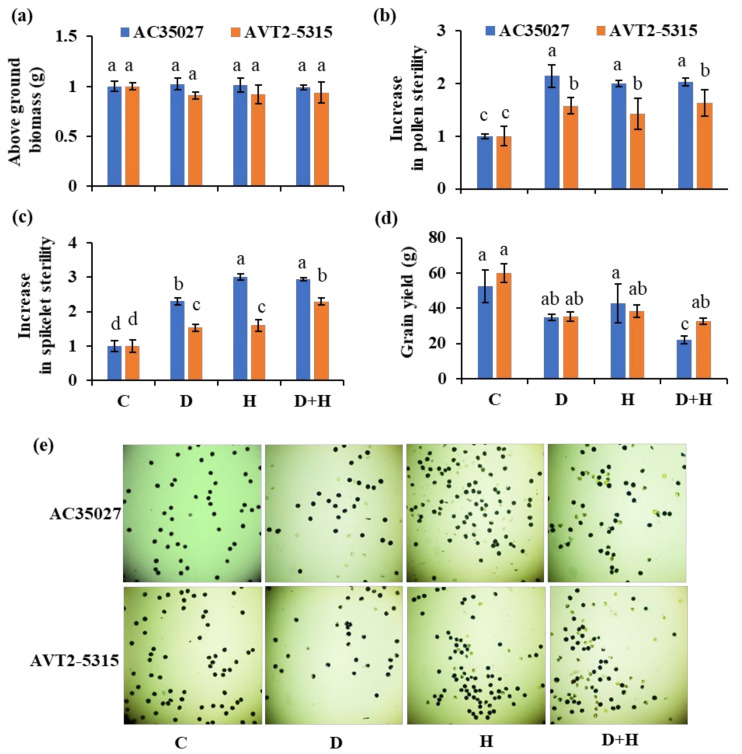
Biomass and yield parameters under stress in contrasting cultivars. Aboveground biomass (**a**); pollen sterility (**b**); spikelet sterility (**c**); grain yield (**d**) and pollen phenotype (**e**) of rice plants under control (C), drought (D), heat (H) and combined stress (D+H) at anthesis. Data are the mean values ± SE (n = 5). Means with the same letter do not differ significantly at *p* ≤ 0.05.

Intrinsic tolerance ability and source strength are the two major traits affecting spikelet fertility and hence yield in rice. Therefore, we measured the biomass, pollen sterility, spikelet sterility, and yield in both the cultivars treated with the individual as well as the combined stresses. The aboveground biomass (exclusive of grain weight), was comparable across both cultivars under all treatments (Figure 2a), as stress was given when both the cultivars reached their maximum growth, at anthesis. The leaf weight was also comparable under all treatments in both the cultivars, suggesting a similar source strength in both the cultivars (data not shown). Both pollen and spikelet sterility increased under all stresses in both the cultivars, with AC35027 showing more sterility compared to AVT2-5315 (Figure 2b,c). A 2-fold and ~1.5-fold increase in pollen sterility was observed in AC35027 and AVT2-5315, respectively, under all stress treatments (Figure 2b,e). Similarly, a greater than two-fold increase in spikelet sterility was observed under all stress treatments compared to controls in AC35027 (Figure 2c). Whereas, in AVT2-5315, only under combined stress spikelet sterility was more than two-fold. Among the stresses, combined stress caused more spikelet sterility in both cultivars. Under combined stress, AVT2-5315 also maintained a grain yield similar to individual stress; however, a greater decline in yield of 50% was seen in AC35027 under combined stress (Figure 2d). These results suggest a greater effect of combined stress on sterility and yield in AC35027 compared to AVT2-5315, and that this is due to potential differences in intrinsic tolerance abilities rather than source limitation, as both the cultivars had similar biomass and yield potential under control conditions.

### 2.3. Cultivars Showed Differential Accumulation of Phytohormones under Stress

Phytohormones play a significant role in controlling spikelet fertility and sink drawing ability under stress as well as control conditions. To understand the phytohormonal regulation under individual as well as combined stress, a targeted phytohormone profiling was performed using LC–MS/MS, and fifteen phytohormones were quantified from flag leaf and spikelet tissues. Principal component analysis (PCA) performed on the control and stress data in flag leaf and spikelet showed a clear separation between the two tissues, with a few common hormones shared between the tissues of each cultivar (Figure S3). Hormones from spikelets were separated by principal component 1 (PC1), which explained 32.8% of the total variance in the dataset, while the hormones from flag leaf were separated by PC2, explaining a further 14.4% of the variance (Figure S3).

**Figure 3:**
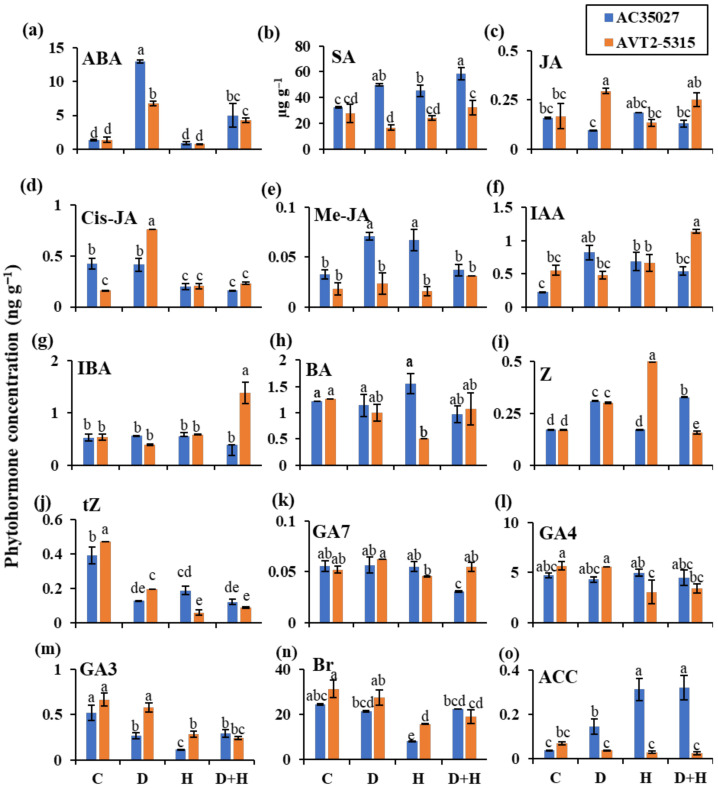
The phytohormone concentration in flag leaves of contrasting rice cultivars under stress at anthesis. Abscisic acid (**a**); salicylic acid (**b**); jasmonic acid (**c**); cis-jasmonate (**d**); methyl–jasmonate (**e**); indole–3–acetic acid (**f**); indole–3–butyric acid (**g**); 6–benzylaminopurine (**h**); zeatin (**i**); trans–zeatin (**j**); gibberellic acid 7 (**k**); gibberellic acid 4 (**l**); gibberellic acid 3 (**m**); 24–epibrassinolide (**n**) and 1–aminocyclopropane–1–carboxylic acid (**o**). Data are the mean values ± S.E (n = 3). Means with the same letter do not differ significantly at *p* ≤ 0.05. C: control; D: drought; H: heat and D+H: combined drought and heat stress.

In flag leaves, there was no significant difference in hormonal accumulation under control conditions between the two cultivars, except cis-jasmonate (cis-JA), which accumulated more in AC35027. Under combined stress, AC35027 showed higher accumulation of ABA (2.68-fold), SA (0.8-fold), zeatin (0.94-fold), and 1-amino cyclopropane-1-carboxylic acid (ACC, 7-fold) compared to the control condition (Figure 3). There was also a decrease in the accumulation of cis-JA (−0.62-fold), trans-zeatin (−0.69-fold), GA7 (−0.50-fold) and GA3 (−0.44-fold) in AC35027 compared to the control condition. On the other hand, in AVT2-5315, a significant increase in the accumulation of ABA (2.05-fold), IAA (1.07-fold) and IBA (1.6-fold) was observed under combined stress compared to the control condition (Figure 3). Similar to AC35027, there was also lower accumulation of trans-zeatin (−0.92-fold), GA3 (−0.64-fold) along with 24-epi-brassinolide (Br, −0.39-fold) in AVT2-5315. Among the cultivars, AVT2-5315 accumulated significantly higher concentrations of JA, IAA, IBA, GA7 and lower concentrations of SA, zeatin and ACC under combined stress compared to AC35027 (Figure 3). Under drought stress, AC35027 showed higher accumulation of ABA (8.59-fold), SA (0.53-fold), Me-JA (1.33-fold), IAA (2.57-fold), zeatin (0.82) and ACC (2.75-fold) and lower accumulation of trans-zeatin (−0.67) and GA3 (−0.48) compared to the control conditions (Figure 3). In AVT2-5315, an increase in ABA (2.05-fold), JA (0.76-fold), cis-JA and zeatin (0.76-fold) and decrease in trans-zeatin (0.4-fold) was observed compared to the control condition (Figure 3). Under heat stress, in AC35027, there was increased accumulation of SA (0.39-fold), Me-JA (1.33-fold), IAA (1.96-fold) and ACC (6.75-fold), while there was decreased accumulation of cis-JA (−0.52-fold), trans-zeatin (−0.51-fold), GA3 (−0.79-fold) and Br (−0.67-fold) compared to the control condition. In AVT2-5315, zeatin (1.94-fold) increased while BA (−0.26-fold), trans-zeatin (−0.95-fold), GA4 (−0.46-fold), GA3 (−0.58-fold) and Br (−0.49-fold) reduced compared to the control condition (Figure 3). Together, SA, Me-JA, and ACC accumulated relatively highly in AC35027 under all stresses compared to controls. Under combined stress, the levels of IAA and IBA in AVT2-5315 and ACC in AC35027 were relatively higher.

**Figure 4:**
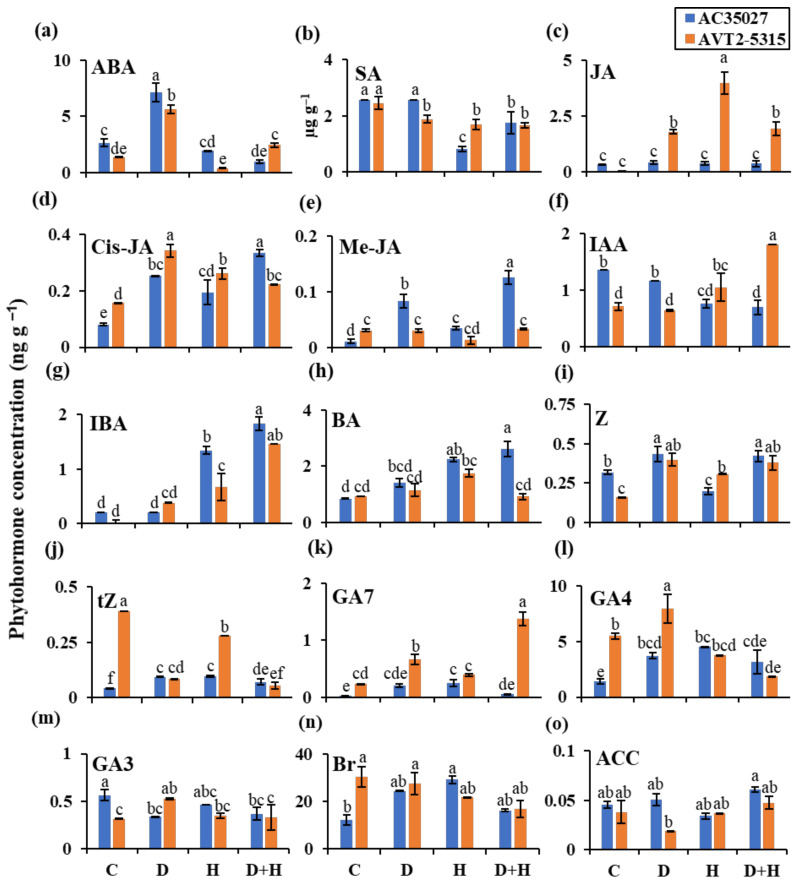
The phytohormone concentration in spikelets of contrasting rice cultivars under stress at anthesis. Abscisic acid (**a**); salicylic acid (**b**); jasmonic acid (**c**); cis–jasmonate (**d**); methyl–jasmonate (**e**); indole–3–acetic acid (**f**); indole–3–butyric acid (**g**); 6–benzylaminopurine (**h**); zeatin (**i**); trans–zeatin (**j**); gibberellic acid 7 (**k**); gibberellic acid 4 (**l**); gibberellic acid 3 (**m**); 24–epibrassinolide (**n**) and 1–aminocyclopropane–1–carboxylic acid (**o**). Data are the mean values ± SE (n = 3). Means with the same letter do not differ significantly at *p* ≤ 0.05. C: control; D: drought; H: heat and D+H: combined drought and heat.

In spikelet tissue, a large variation in hormonal accumulation was observed even under control condition (Figure 4). There was a relatively higher accumulation of ABA, IAA, zeatin, and GA3 in AC35027, and cis-JA, Me-JA, trans-zeatin, GA7, GA4 and Br in AVT2-5317 (Figure 4). Under combined stress, there was an increase in the accumulation of cis-JA (3.13-fold), Me-JA (12-fold), IBA (6.6-fold), BA (2.05-fold), zeatin (0.31-fold) and trans-zeatin (0.75-fold) in AC35027 and ABA (0.79-fold), JA (63.33-fold), cis-JA (0.38-fold), IAA (5.83-fold), IBA 145-fold), zeatin (1.38-fold) and GA7 (4.71-fold) in AVT2-5315 compared to the control condition (Figure 4). Furthermore, there was a decrease in the accumulation of ABA (−0.63-fold), SA (−0.61-fold), IAA (−0.68-fold) and GA3 (−0.34-fold) in AC35027, and SA (−0.32-fold), trans-zeatin (−0.93-fold) and GA4 (−0.66-fold) in AVT2-5315 compared to control. Among the cultivars, in AC35027, cis-JA, Me-JA, and BA accumulated in higher quantities compared to AVT2-5315. Similarly, in AVT2-5315, ABA, JA, IAA, and GA7 accumulated in higher concentrations than AC35027 (Figure 4). Under drought stress, there was increased accumulation of ABA (1.69-fold), cis-JA (3.13-fold), Me-JA (12-fold), zeatin (0.31-fold), trans-zeatin (0.75-fold), GA4 (1.22-fold) and Br (0.32-fold), and decreased accumulation of GA3 (−0.34-fold) compared to the control condition in AC35027 (Figure 4). Similarly, there was increased accumulation of ABA (3.09-fold), JA (58.67-fold), cis-JA (1.13-fold), IBA (37-fold), zeatin (1.5-fold), GA7 (1.79-fold), GA4 (0.45-fold) and GA3 (0.63-fold), and there was decreased accumulation of SA (−0.23-fold) and trans-zeatin (−0.89-fold) in AVT2-5315. Under heat stress, an increase in cis-JA (1.4-fold), Me-JA (2-fold), IBA (5-fold), BA (1.6-fold), trans-zeatin (1.5-fold), GA7 (7-fold), GA4 (2-fold), and Br (1.4-fold) was observed in spikelets of AC35027 (Figure 4). There was also decreased accumulation of SA (−0.81-fold), IAA (−0.64-fold) and zeatin (−0.38-fold) in AC35027 compared to control condition. Similarly, an increased accumulation of JA (131-fold), cis-JA (0.63-fold), IAA (0.48-fold), IBA (66-fold) and zeatin (0.94-fold), and decreased accumulation of SA (−0.31-fold) and trans-zeatin (−0.31-fold) was observed in spikelets of AVT2-5315 compared to the control. Together, JA and GA7 in AVT2-5315 and Me-JA and BA in AC35027 showed relatively higher accumulation under all stresses. Under combined stress, JA and GA7 in AVT2-53715 and Me-JA in AC35027 accumulated relatively higher.

### 2.4. Key Biosynthetic Pathway Gene Expression Validates the Differential Accumulation of Hormones

**Figure 5:**
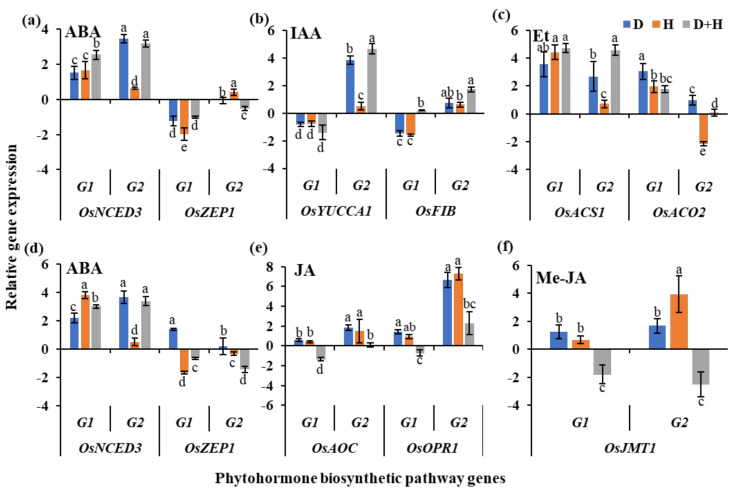
Expression of key phytohormone biosynthetic pathway genes in flag leaves and spikelets of contrasting rice cultivars under stress. Expression of abscisic acid (**a**); auxin (**b**) and ethylene (**c**) biosynthetic pathway genes in flag leaves, and expression of abscisic acid (**d**); jasmonic acid (**e**) and methyl-jasmonate (**f**) biosynthetic pathway genes in spikelets. The expressions are represented as log_2_ fold change under drought (D), heat (H) and combined (D+H) stress over control normalized with reference gene *ubiquitin*. The data are mean values ± SE (n = 3). Duncan’s Multiple Range Test was performed separately for each gene represented by hormone type. G1: AC35027 and G2: AVT2-5315.

The expression of key phytohormone biosynthetic pathway genes was investigated to validate the differential accumulation of phytohormones under stress. As a stress marker ABA biosynthetic pathway gene, *9-cis-epoxycarotenoid dioxygenase 3* (*OsNCED3*), showed increased expression under drought as well as combined stress in both the cultivars and both the tissues (Figure 5a,d). However, *zeaxanthin epoxidase 1* (*OsZEP1*), another ABA biosynthetic pathway gene, showed decreased expression in both cultivars and tissues. In flag leaves, auxins, IAA and IBA, accumulated in higher concentrations in AVT2-5315. Therefore, the expression of key auxin biosynthetic pathway genes, *OsYUCCA1*, encoding flavin monooxygenase-like enzyme, and *OsFIB*, encoding tryptophan aminotransferase, were analyzed. Increased expression of both *OsYUCCA1* and *OsFIB* in AVT2-5315 and reduced expression of both the genes in AC35027 were observed under combined stress (Figure 5b). In AC35027, increased accumulation of ET was observed under combined stress. Therefore, expression of key ET biosynthetic pathway genes, *OsASC1* and *OsACO2* were analyzed. Results showed increased transcript levels of both the genes in AC35027 while in AVT2-5315, *OsACS1* transcript levels increased and the marginal increase in expression of *OsACO2* was observed under combined stress (Figure 5c). Together, these results validate the increased expression of auxin and ET biosynthetic pathway genes in AVT2-5315 and AC35027, respectively.

In spikelets, under combined stress, AVT2-5315 showed higher accumulation of JA and AC35027 showed higher accumulation of Me-JA. Therefore, the expression of key JA biosynthetic pathway genes, *OsAOC* and *OsOPR1* and key Me-JA biosynthetic pathway gene, *jasmonic acid carboxyl methyltransferase 1* (*OsJMT1*) were analyzed. In AVT2-5315, under all stresses, both *OsAOC* and *OsOPR1* showed increased transcript levels with only a marginal increase in transcripts of *OsAOC* under combined stress (Figure 5e). However, under combined stress, transcript levels of both the genes were reduced in AC35027. The expression of *OsJMT1* was higher in both the cultivars under individual drought and heat stress (Figure 5f). However, under combined stress its expression was reduced in both the cultivars. Together, our results validate the increased expression of *OsAOC* and *OsOPR1* in AVT2-5315, along with the increased accumulation of JA.

## 3. Discussion

Water scarcity under the changing climate is forcing rice cultivation under rainfed and semi-irrigated ecosystems. Under these conditions, rice will more often be exposed to a combination of drought and heat stresses. Though the effects of combined drought and heat stress at the vegetative stage are minimal, stress at the reproductive stage will significantly reduce yield [7,8,12,30]. Therefore, improving rice tolerance to combined stress gains significance, especially under the changing climate. Efforts are being made to identify and incorporate stress escape or avoidance traits, such as early morning flowering, to minimize exposure of rice to severe environmental conditions at sensitive stages [31]. However, the intrinsic ability of a genotype/cultivar to tolerate stress, leading to better yield, will have broader implications towards developing climate-resilient rice. In this study, two rice cultivars, AVT2-5315 and AC35027, with contrasting spikelet fertility under combined drought and heat stress were selected for further physiological characterization. Except for Fv′/Fm′ under drought, there were no significant differences in RWC, A, gs, Fv′/Fm′ and biomass under individual as well as combined stress between the cultivars (Figure 1). However, both the cultivars significantly differed in pollen and spikelet sterility under individual and combined stresses (Figure 2b,c). Though grain yield did not differ among cultivars under individual stresses, AVT2-5315 showed better yield under combined stress, suggesting its intrinsic tolerance ability due to better pollen and spikelet fertility (Figure 2d). Similar observations were made by Li et al. [11], with combined stress significantly reducing anther dehiscence, pollen number, germination, and spikelet fertility in susceptible Moroberekan compared to tolerant Nagina 22. Though pollen sterility did not differ between the stresses in AC35027, spikelet sterility increased under heat and combined stress, suggesting the possible dominance of heat stress as well as asynchrony in pollen dehiscence and/or stigma receptivity. Similar observations were made by Rang et al. [8], with heat stress dominantly reducing spikelet fertility under combined stress in Moroberekan. In AVT2-5315, pollen sterility did not significantly differ across the stresses. However, spikelet sterility increased under combined stress with no significant difference between drought and heat, suggesting the cumulative effect of drought and heat stresses when combined. Nevertheless, it maintained a similar yield across the stresses. Due to its intrinsic tolerance ability, AVT2-5315 can serve as a good genetic source for further understanding of combined stress tolerance in rice at the reproductive stage.

Phytohormones are called the watchdogs of plant stress responses and their orchestrated crosstalk regulates several processes leading to plant stress adaptation [32]. Therefore, it is interesting to profile the phytohormones in rice under combined stress, especially at the reproductive stage, to understand and improve the combined stress tolerance of rice through the modulation of phytohormones. To the best of our knowledge, this is the first report of phytohormone profiling in rice flag leaf and spikelets under combined drought and heat stress. Targeted profiling of 15 phytohormones in flag leaf and spikelets in two cultivars and four treatments was performed using LC–MS/MS. PCA showed clear separation of flag leaves and spikelet tissues, suggesting tissue-specific regulation of hormonal biosynthesis (Figure S3). Under combined stress, in flag leaves, the low sterility cultivar, AVT2-5315, accumulated higher auxins, IAA and IBA (Figure 3f,g)—while in the high sterility cultivar, AC35027, there was a higher accumulation of ET (Figure 3c). IAA is the major endogenous auxin in plants and IBA is converted to IAA in a peroxisomal β-oxidation process. IAA has been suggested to be the key regulator of growth and development in plants, including rice [33]. However, altered accumulation of IAA has been shown in rice under drought and heat stress. For example, there was a reduced level of endogenous IAA in rice seedlings under drought stress [34]. However, under heat stress, IAA levels increased. Therefore, the increased levels of IAA under combined stress in the flag leaves of AVT2-5315 suggests the plausible dominance of heat stress, and hence, reduced sterility. Exogenous application of IAA has been shown to protect spikelet fertility and grain yield under drought and heat stresses in rice [35]. Among the IAA biosynthetic enzymes identified in rice, the TAA family of aminotransferases and the YUCCA family of flavin-containing monooxygenases are the most important. Though these enzymes are encoded by several aminotransferases and YUCCA family genes, under stress, *OsFIB* and *OsYUCCA1* have been shown to be predominant [36,37]. Under drought stress, the expression of both the genes was reduced, while there was increased expression of *OsFIB* under heat stress [35,36,37]. In our study, both *OsYUCCA1* and *OsFIB* expression increased under individual drought, heat and combined stress in flag leaves of the low sterility cultivar, AVT2-5135 (Figure 5b). In corroboration, there was an increased accumulation of auxins (Figure 3f,g). However, in the high sterility cultivar, AC35027, there was down-regulation of both the genes under individual as well as combined stresses (Figure 5b). The role of the phytohormone ET under abiotic stress is well established in submergence tolerance [38]. However, under drought and heat stress, there is no clear evidence to show either the positive or negative role of ET. In our study, there was a relatively higher accumulation of ET in the high sterility cultivar, AC35027 (Figure 3o). Two key steps in the synthesis of ET are the conversion of S-adenosylmethionine to ACC by ACC synthase (ACS), and then oxidation of ACC by ACC oxidase (ACO) to generate ET [39]. Under stress, *OsACS1* and *OsACO2* have been shown to be the key genes and expressed in shoot apical meristems, panicles, and developing seeds [40]. In our study, *OsACS1* transcript levels were higher under all stresses in both the cultivars (Figure 5c). However, *OsACO2* transcripts were higher in the high sterility cultivar, AC35027, under all stresses, while in AVT2-5315, its transcripts increased only under drought but reduced under heat with no change under combined stress. At least in AC35027, increased accumulation of ET is in corroboration with increased expression of both key biosynthetic pathway genes.

In spikelets, under combined stress, JA and Me-JA showed relatively higher accumulation in low and high sterility cultivars, respectively (Figure 4c,e). JA plays an important role in regulating development and growth under stress as well as control conditions in plants [41,42]. The most prominent role of JA in plants has been suggested to be the regulation of reproductive organ differentiation and fertility [43]. However, the methyl ester of JA, Me-JA, has been shown to play a negative role in the reproductive growth of rice. Higher levels of Me-JA have been shown to reduce spikelet numbers and hence yield in rice [44]. In rice, *OsAOC*, encoding allene oxide cyclase, and *OsOPR1*, encoding 12-oxophytodienoic acid reductase 1, have been suggested to be altered under stress [45,46,47]. In our study, under combined stress, there was relatively higher expression of these genes in low sterility cultivar than in high sterility cultivar (Figure 5e), suggesting a high correlation with the JA and transcript levels of its key biosynthetic pathway genes. Though Me-JA accumulated at a higher level in the high sterility cultivar under combined stress (Figure 4e), there was no correlation with transcript levels of its key biosynthetic pathway gene, *OsJMT1*, encoding jasmonic acid carboxyl methyltransferase 1. However, reports suggest that increased accumulation of Me-JA due to overexpression of *JMT1* resulted in reduced spikelet number and yield under drought stress [44]. Though there are controversial reports on the role of JAs in rice reproductive growth and development, their levels and interactions with ABA seem to control these processes [25].

In summary, our data show that the contrasting cultivars did not differ in their physiological parameters under all stresses. The low pollen and spikelet sterility, and better yield in AVT2-5315 than AC35027, are due to their intrinsic tolerance ability. Higher auxins in flag leaves and JA in spikelets could have contributed to low sterility in AVT2-5315. On the contrary, high ET and Me-JA would have reduced spikelet fertility and yield in AC35027. However, the role of JAs in regulating reproductive growth in rice under stress and increased accumulation of ET in the high sterility cultivar in the flag leaves and its relevance to increased sterility needs further investigation.

## 4. Materials and Methods

### 4.1. Plant Growth Conditions and Stress Treatments

Rice (*Oryza sativa* L.,) cultivars with contrasting spikelet fertility, AC35027 (high sterility) and AVT2-5315 (low sterility) were grown in 12-inch pots filled with 9 kg of red sandy-loam soil and farmyard manure (3:1). The water-holding capacity of the soil was calculated following the gravimetric method [48]. Two plants were grown in each pot with taps at the bottom to facilitate water control under natural semi-irrigated aerobic conditions until flowering. At anthesis, plants were subjected to drought stress by withholding irrigation till the water content was down to 50% field capacity (FC, i.e., 70–75% leaf RWC) by monitoring the pot weight. Heat stress of 38 °C was given at anthesis for three consecutive days in a temperature-controlled chamber for 6 h (9 a.m. to 3 p.m.). For combined stress, drought-stressed plants at 50% FC were given heat stress of 38 °C for three days while maintaining 50% FC. A set of control plants (100% FC) were maintained under normal growth conditions. Stress treatment and timelines are illustrated in Figure S4. At the end of the stress period, flag leaf and spikelet tissue were harvested and flash-frozen using liquid nitrogen and stored at −80 °C for phytohormone quantification. Care was taken to collect the spikelets at the anthesis stage. The remaining plants were allowed to recover and grow to physiological maturity for recording biomass, spikelet sterility, and grain yield.

### 4.2. Measurement of Physiological Parameters

#### 4.2.1. Relative Water Content 

Leaf samples were taken from five biological replicates in each treatment on the 3rd day of stress treatment and their fresh weight (FW) was recorded immediately. The leaf sample was then incubated in deionized water overnight and the turgid weight (TW) was recorded. Samples were then placed into a brown paper bag and oven-dried at 70 °C for 72 h and the dry weight (DW) taken. The RWC was computed as RWC (%) = (FW−DW)/(TW−DW) × 100.

#### 4.2.2. Gas Exchange Parameters

Photosynthetic rate (A), stomatal conductance (gs), the effective quantum yield of PSII (ΦPSII), photochemical quenching (qP), non-photochemical quenching (qN), and light-adapted chlorophyll fluorescence (Fv′/Fm′) were recorded using a portable infrared gas analyzer (LI-COR 6400, Lincoln, NE, USA). All observations were recorded at a photosynthetic photon flux density (PPFD) of 1500 μmol m^−2^ s^−1^, the reference CO_2_ concentration of 400 ppm, and cuvette temperature of 28 °C with 2.5 cm^2^ leaf area chamber. The flow of dry air was kept at 500 mL min^−1^ to maintain comparable relative humidity inside the leaf chamber. All gas exchange measurements were recorded between 09:00 to 11:00 h (local time) in second fully expanded leaves on the third day of stress.

### 4.3. Yield and Related Parameters

#### 4.3.1. Pollen Sterility

Anthers were collected from control and stressed plants on the third day of stress and stored in 70% ethanol. They were later stained with 1% I_2_KI to assess pollen sterility as described by [49].

#### 4.3.2. Spikelet Sterility and Grain Yield

Panicles were harvested separately from each cultivar and treatment. Filled and unfilled grains for each panicle were weighed, counted and sterility percentage under different treatment conditions was determined. Above-ground biomass (sum of leaf, shoot, and panicle weight) was also determined after drying the samples at 70 °C for 72 h and taking the dry weight.

### 4.4. Targeted Phytohormone Analysis

#### 4.4.1. Standard Preparation

MS/MS conditions were optimized to produce a maximal signal as described by [50]. 1-Amino cyclopropane-1-carboxylic acid (ACC), zeatin trans-isomer (Tr-Z), zeatin (Z), Cis-JA and Me-JA standards were analyzed in positive scan mode as [M + H]^+^ ions; while GA3, GA4, GA7, SA, IAA, ABA, JA, IBA, Br and 6-benzylaminopurine (BA) standards were analyzed in negative scan mode as [M − H]^−^ ions. Precursor and product ions specific for each hormone were identified, using standards and appropriate precursor-to-product ion transitions representing a major fragmentation path unique to each phytohormone (Supplementary Table S1).

#### 4.4.2. Sample Preparation

Hormones from flag leaf and spikelets of rice were analyzed using Liquid Chromatography–Mass Spectrometry (LC–MS) as described by Pan et al. [50]. Tissue (0.5 g) was completely homogenized using 1-propanol, H_2_O and concentrated HCl (2:1:0.002, *v*/*v*/*v*), sonicated for 30 min, and kept overnight at 4 °C. To the homogenate, 10 mL of dichloromethane was added and sonicated again for 30 min. Once sonicated, the samples were centrifuged at 12,000 rpm for 10 min and the bottom layer was transferred to a conical flask containing sodium sulphate (anhydrous) to remove traces of water from the sample followed by flash evaporation. After complete drying, the sample was redissolved in 1 mL of methanol and 0.05% formic acid (1:1, *v*/*v*) and stored at 4 °C. Before loading, the sample was dried using N_2_ gas and redissolved in acetonitrile: water (95:5), filtered using a nylon filter paper, and injected into LC–MS (Waters, Milford, MA, USA) for further analysis. LC–MS was equipped with a reversed-phase column (UPLC BEH-C18 column, 2.1 × 50 mm × 1.7 μm, protected by a guard column, BEH C-18 vanguard, 2.1 × 5 mm × 1.7 μm), maintained at a column temperature of 25 °C, using a binary solvent system composed of (A) water: acetonitrile: acetic acid (95:5:0.05) and (B) acetonitrile: water: acetic acid (95:5:0.05) as a mobile phase. A flow rate of 0.2 mL/min was maintained and separations were performed using a gradient method for 20 min per sample. The initial gradient was composed of 85% solvent A and 15% of solvent B (1 min). At the 12th min, the gradient was changed to 15% of solvent A and 85% of solvent B (1 min) and at the 14th min, a linear gradient of 85% solvent A and 15% solvent B (0.5 min). The system was then returned to the initial conditions at the 15th min and equilibrated for 1 min before the next injection. The data was acquired using the Acquity H software (Waters, Milford, MA, USA).

### 4.5. Gene Expression Analysis

Total RNA was isolated using the TRIzol method (Invitrogen, Carlsbad, CA, USA) from the flag leaf and spikelet tissues as per the supplier’s instructions. The cDNA synthesis was done using an RT-PCR kit (GeNeiTM M-MuLV RT-PCR Kit, Genei Laboratories Pvt. Ltd., Bangalore, India), following the supplier’s protocol. The cDNA was used as the template for expression analysis of key hormone biosynthetic pathway genes. Quantitative RT-PCR (qRT-PCR) analysis was performed using the Bio-Rad CFX96 C1000 Touch™ (CT035400, Bio-Rad, Hercules, CA, USA) with iTaq UniverSYBR Green SMX 1000 (Bio-Rad, Hercules, CA, USA). *Ubiquitin* was used as an endogenous reference gene to normalize the data. The relative expression of genes was calculated using the 2^−^^ΔΔCt^ method [51] and represented as log_2_ fold-change. The genes and the primers used in the study are given in Supplementary Table S2.

### 4.6. Statistical Analysis

Duncan’s multiple range test was performed using Microsoft Excel XLSTAT (version 2019) to determine the significance (*p* ≤ 0.05) between treatment groups. Multivariant analysis was performed using the MetaboAnalyst 5.0 web tool. The data are presented as mean values ± standard error (SE).

## Data Availability

Available within the manuscript and supplementary information.

## Supplementary Materials

The following are available online at https://www.mdpi.com/article/10.3390/ijms222111690/s1.
