# Supplementary material for: Targeted Phytohormone Profiling Identifies Potential Regulators of Spikelet Sterility in Rice under Combined Drought and Heat Stress
Source: Int J Mol Sci. 2021 Oct 28;22(21):11690. doi: 10.3390/ijms222111690 (PMC8584246; doi:10.3390/ijms222111690)
Supplement: Supplementary file 1 [file ijms-22-11690-s001.zip › ijms-1395213-supplementary.pdf]

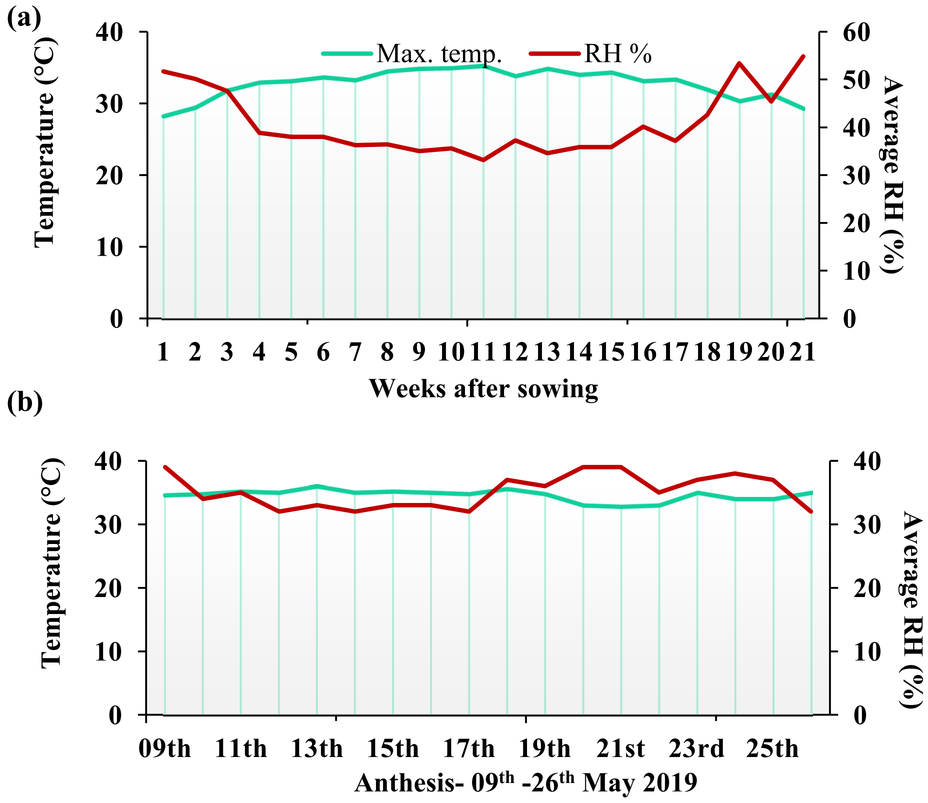

Figure S1. Temperature and relative humidity at the site during the experiment. The meteorological observation [Lat:13° 05′ N; Long: 77° 34′ E and Alt.: 924 m (amsl)] for the entire growth period from February to May 2019 (**a**) and during the anthesis period from 9th-26th May 2019 (**b**).

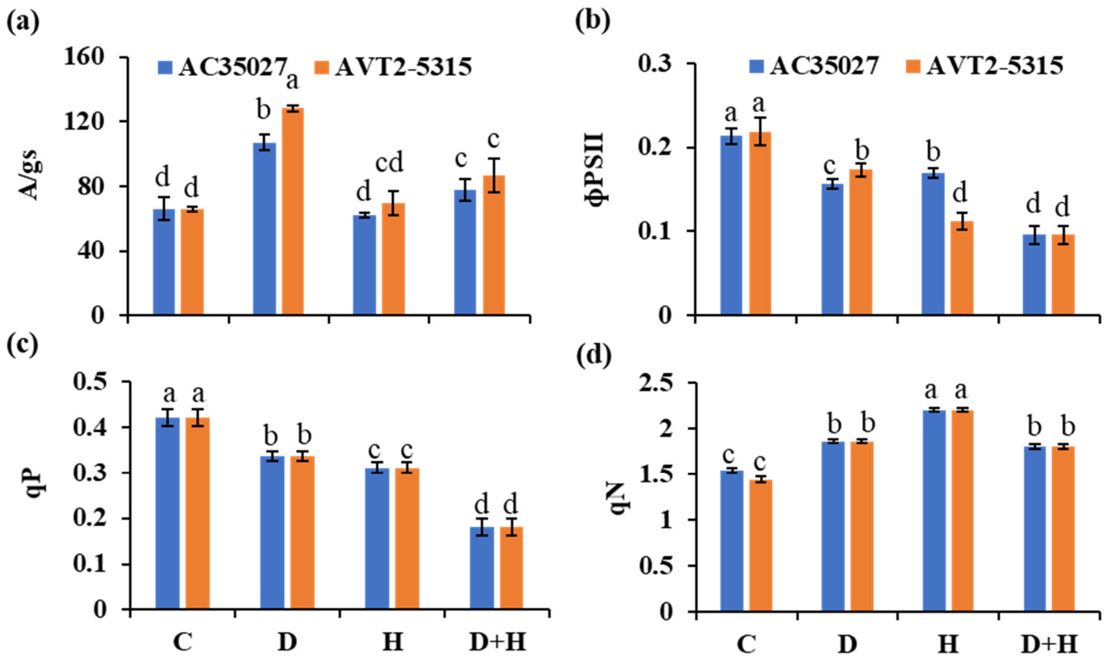

Figure S2. Other physiological parameters affected under stress. Intrinsic water use efficiency (**a**); Effective quantum yield of photosystem II (**b**); Photochemical quenching (**c**) and Non-photochemical quenching (**d**) of rice plants treated with control (C), drought (D), heat (H) and combined stress (D+H) at anthesis. Data are mean values ± SE (n = 5). Means with the same letter do not differ significantly at $p \leq 0.05$.

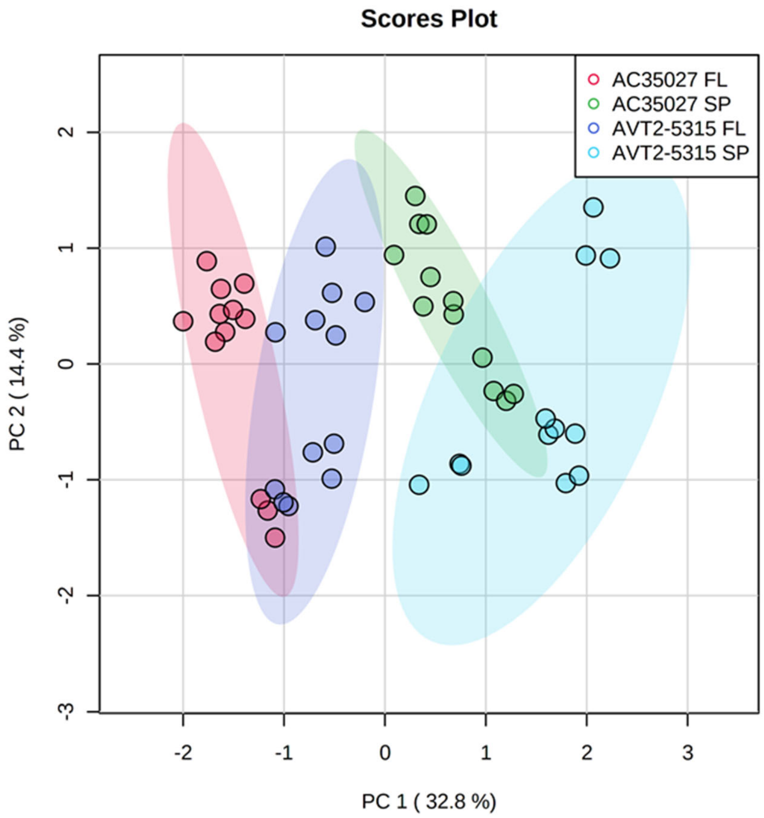

Figure S3. Score plot from the PCA of rice phytohormone profiles. The first 2 principal components (PC1 and PC2) are shown for samples from flag leaves (FL) and spikelets (SP) collected under control, drought, heat and combined stress conditions from the rice cultivars AVT2-5315 and AC35027. Scores are means of the median-normalized and log-transformed mass spectral intensities of 15 phytohormones detected in common across both genotypes and tissues.

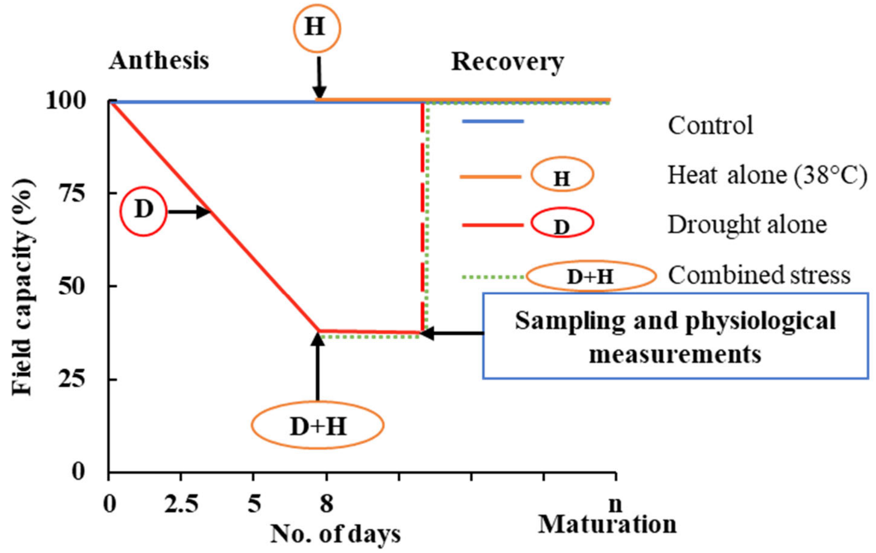

Figure S4. Schematic illustration of stress imposition. Plants were exposed to heat (H), drought (D) and combined (D+H) stress at anthesis stage for three consecutive days. Drought stress was 50% FC, heat stress was 38 °C and combined stress was 50% FC and 38 °C. Physiological measurements and sample collections were done on the third day of stress, after which, the plants were allowed to recover until physiological maturity.

Supplementary Table S1. Optimized MS/MS conditions for the analysis of plant hormones

Sr. No.	Hormone	Abbreviation	Mode (+/−)	Mass	*Rt* (min)	Precursor ion (*m*/*z*)	Product ion (*m*/*z*)	Cone voltage (V)	Collision energy (eV)
1	1-Amino cyclopropane-1-carboxylic acid	ACC	+	101.0	0.61	102.03	56.01	10	8
2	Trans zeatin riboside	Tr-Z	+	351.1	0.81	352.2	220.21	36	22
3	Zeatin	Z	+	219.1	0.70	220.13	136.03	18	14
4	Cis-jasmonate	Cis-JA	+	164.0	8.33	165.03	147.02	10	8
5	Methyl-jasmonate	Me-JA	+	224.0	8.70	225.03	147.00	8	12
6	Gibberellic acid 3	GA3	−	345.9	0.94	345.00	143.03	22	20
7	Salicylic acid	SA	−	138.0	2.95	137.03	93.02	14	8
8	Indole-3-acetic acid	IAA	−	175.0	3.83	173.97	130.04	14	10
9	Abscisic acid	ABA	−	264.0	4.76	263.22	153.15	20	8
10	Jasmonic acid	JA	−	210.0	6.32	208.97	59.01	18	10
11	Indole-3-butyric acid	IBA	−	203.0	6.54	201.90	157.95	22	10
12	Gibberellic acid 7	GA7	−	331.0	7.32	329.14	223.20	32	12
13	Gibberellic acid 4	GA4	−	332.0	7.42	331.03	213.04	30	24
14	24-Epibrassinolide	Br	−	480.3	7.92	479.35	129.12	50	20
15	6-Benzyl adenine	BA	−	225.2	3.21	224.16	133.12	30	20

Supplementary Table S2. List of phytohormone biosynthetic pathway genes and primers used in the study

Hormone	Key genes in the biosynthetic pathway	Primers	Oligo (5'-3')
ABA	*OsNCED3*	Os03g0645900_F	CCCCTCCCAAACCATCCAAACCGA
		Os03g0645900_R	TGTGAGCATATCCTGGCGTCGTGA
	OsZEP1	Os04g0448900_F	GCACGCTCCCATATGAATCT
		Os04g0448900_R	GCTGTACAGTAGCTGATGAGTG
JA	*OsAOC*	Os03g0438100_F	ATGTGCGGTGTGTATCGTATC
		Os03g0438100_R	CCAATCCACCTACTACCAAGAAG
	OsOPR1	Os06g0216300_F	CACATGGTGGAGCCAAGAA
		Os06g0216300_R	CAGCGATGAAAGTGCCATTG
IAA	*OsYUCCA1*	Os01g0645400_F	TAAGGTAGTGGGAGCAGTGA
		Os01g0645400_R	GCCTGTGGCGAGGATTATT
	OsFIB	Os01g0169800_F	TCTGCAACTTCACCAAGGAG
		Os01g0169800_R	CTTGTGCCCACGGAGAAA
Me-JA	*OsJMT1*	Os05g0102000_F	AGACAAGCAGCACCATCAG
		Os05g0102000_R	GTGGCGGTAGTAATTGTGGA
ET	*OsACS1*	Os03g0727600_F	GGTCTCGGATGATCACATATCG
		Os03g0727600_R	AGGTTACAACAACTGGGAGAAA
	OsACO2	Os09g0451000_F	CGTCTCGTCACCTTGGATAAT
		Os09g0451000_R	AGGACAGCCGACAAAGATAC
	Ubiquitin (reference gene)	Os03g0234200_F	CGCAAGTACAACCAGGACAA
		Os03g0234200_R	GCTGTGACCACACTTCTTCTT
